# Factors Affecting the Rate of Colonoscopy Among African Americans Aged Over 45 Years

**DOI:** 10.7759/cureus.46525

**Published:** 2023-10-05

**Authors:** Afomachukwu Ajufo, Aisha O Adigun, Majed Mohammad, Juliet C Dike, Abidemi O Akinrinmade, Temitayo M Adebile, Chioma Ezuma-Ebong, Kafayat Bolaji, Okelue E Okobi

**Affiliations:** 1 Internal Medicine, All Saints University School of Medicine, Roseau, DMA; 2 Infectious Diseases, University of Louisville, Louisville, USA; 3 Geriatrics, Mount Carmel Grove City Hospital, Grove City, USA; 4 Internal Medicine, University of Calabar, Calabar, NGA; 5 Medicine and Surgery, Benjamin S. Carson School of Medicine, Babcock University, Ilishan-Remo, NGA; 6 Public Health, Georgia Southern University, Statesboro, USA; 7 Nephrology, Boston Medical Center, Malden, USA; 8 Internal Medicine, Angelic Care Hospital, Abuja, NGA; 9 Ophthalmology, Aminu Kano Teaching Hospital, Kano, NGA; 10 Family Medicine, Larkin Community Hospital Palm Springs Campus, Miami, USA; 11 Family Medicine, Medficient Health Systems, Laurel, USA; 12 Family Medicine, Lakeside Medical Center, Belle Glade, USA

**Keywords:** racial disparities, colonoscopy utilization, african americans, colorectal cancer screening, colonoscopy

## Abstract

African Americans continue to have a low rate of colonoscopy screening despite the U.S. Preventive Services Taskforce's (USPSTF) recommendations and its proven benefits. Colonoscopy has proven to be an effective screening and therapeutic procedure. Understanding the root cause of the problem is a crucial step toward achieving the desired colonoscopy rate among this population. This paper evaluates factors that contribute to the underutilization of colonoscopy. The paper also analyzes strategies that could be maximized to increase colonoscopy rates, minimize colorectal cancer inequalities, and promote optimal colorectal health among African Americans.

## Introduction and background

Colorectal cancer (CRC) is a serious public health issue in the United States and one of the leading causes of morbidity and mortality. Despite its decreasing frequency over time, CRC remains the third most common cancer in men and women in the United States. Only patients aged 55 and above have seen a decline in incidence, whereas those aged 40-54 have had an increase of 0.5%-1.3% [1].

The role of screening is crucial because it has the potential to reduce cancer mortality, morbidity, and unnecessary treatment costs by discovering significant lesions before they spread. The U.S. Preventive Services Task Force (USPSTF) recommends screening for people aged 45-75 and recommends that doctors only offer colonoscopies to those aged 76-85 in specific circumstances [2].

According to the latter recommendations, screening everyone in this age group would have a minimal net benefit. Furthermore, some studies suggest that the complications of a colonoscopy, such as bleeding and perforation, increase with age. When determining whether the procedure is appropriate in a given situation, patients and physicians should evaluate the patient's overall health, previous screening history, and preferences [2].

While there are multiple screening techniques available for the prevention of CRC, the most recommended approach is a screening colonoscopy, which is thought to have decreased the occurrence and mortality rates of CRC by 77% and 65%, respectively [3].

Even though colonoscopies are readily available throughout the United States, a study by Adam et al. revealed that African Americans are less likely to be checked for CRC and have higher incidence and mortality rates than White counterparts, despite the well-documented benefits of colonoscopy. New research has shown that physician mistrust may be influencing discrepancies in the use of colonoscopies among African Americans, particularly among African American men. It has also been connected to several co-occurring behavioral and socioeconomic determinants of colonoscopy use, underlying genetic characteristics, educational access, and behavioral and social difficulties [4].

When compared to other ethnicities, African Americans are thought to be the most affected by colon cancer, having the greatest mortality rates. Between 2013 and 2017, there were 43.6 cases per 100,000 African Americans, according to reports. This is significantly higher than the rate among American Indian adults, which was 39.0 cases per 100,000. It is also greater than the incidence among White adults, which was 37.8 instances per 100,000. There were 18.0 deaths per 100,000 people between 2014 and 2018. This is significantly higher than the rates for American Indian and White adults, which were 15.1 and 13.6, respectively [2].

Several recommendations have been made for issuing specific guidelines on colonoscopy to African American adults. The American College of Gastroenterology (ACG) and the American Society of Gastrointestinal Endoscopists (ASGE) both recommend that CRC screening in African Americans should begin at age 45. As a part of their best practice advice, the American College of Physicians has also recommended a screening age of 40 years for African Americans. Due to limited data and evidence, the success of these recommendations cannot be ascertained. Our study aims to explore possible disparity factors, disclose the integrative nature of disparities (environmental, genetic, and behavioral) and their relationship to health outcomes, and offer some long-term solutions to improve the utilization of colonoscopy in African Americans.

## Review

CRC and screening guidelines

Screening has been useful in the downward trend of incidence and mortality of CRC noted over the last two decades. The screening methods available are of great use because of their ability to detect pathologies at different stages [5]. A screening test is high in sensitivity, highly accurate, precise, affordable, and easily accessible. There are a variety of CRC screening methods.

There are currently multiple screening methods including flexible sigmoidoscopy (FS), computed tomography colonography (CTC), fecal occult blood test (FOBT), fecal immunochemical test (FIT), and multitarget stool DNA testing, all with varying indications and timelines. Colonoscopy is generally the preferred option for screening [3,5].

Currently, most U.S. guideline organizations, including the USPSTF, agree that the recommended options in screening for CRC include colonoscopy every 10 years, CTC or sigmoidoscopy every five years, and annual high-sensitivity guaiac-based FOBT (HSgFOBT) or FIT. The American Cancer Society has recommended an interval of three years for multitarget stool DNA testing [5,6]. However, there are variations to these recommendations. For example, in patients with a family history of familial adenomatous polyposis (FAP), screening should start at age 40 or 10 years before the diagnosis of the youngest first-degree relative (FDR). Colonoscopy in these patients should be performed every five years [7]. Additionally, colonoscopy screening should start in patients with inflammatory bowel diseases eight years after disease onset [8]. 

**Figure 1:**
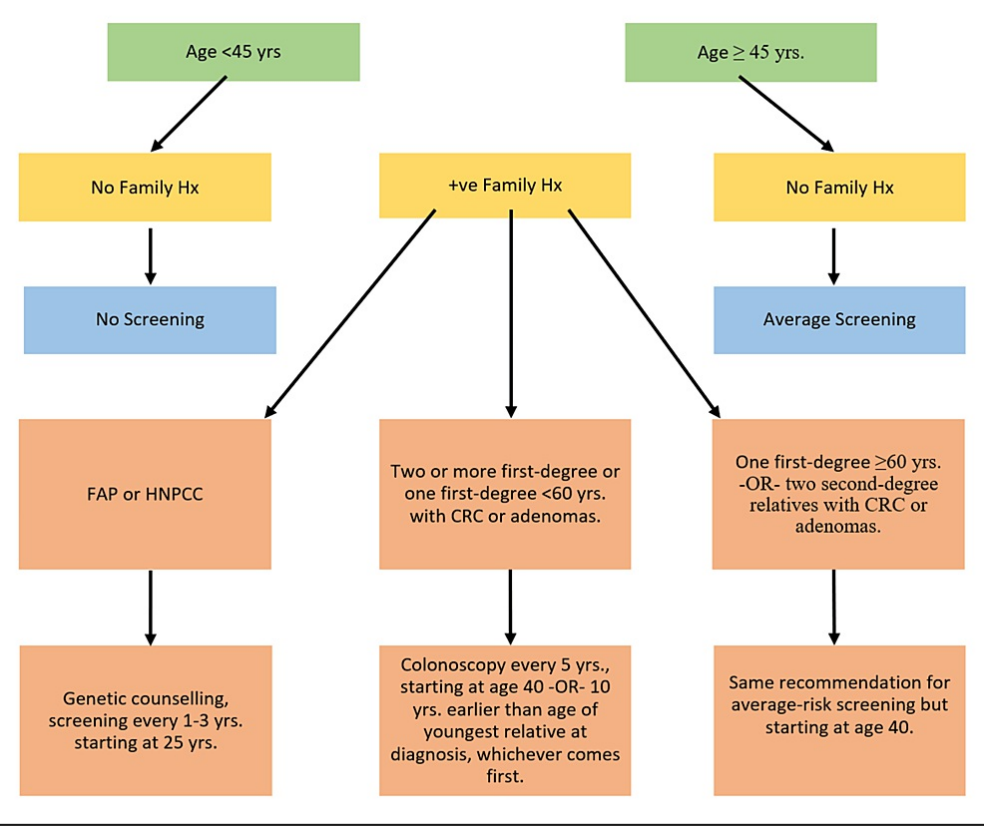
USPSTF's recommendation for colon cancer screening in asymptomatic individuals. USPSTF: U.S. Preventive Services Taskforce; FAP: familial adenomatous polyposis; HNPCC: hereditary nonpolyposis colorectal cancer; CRC: colorectal cancer. Image credit: Authors.

Furthermore, colonoscopy should be done one year after surgery in patients who had proctocolectomy due to CRC and continue every five years thereafter. Additionally, for patients who had proctocolectomy and ileal pouch-anal anastomosis due to CRC or dysplasia, colonoscopy should be performed one year after surgery and continue annually thereafter (Figure 1) [9,10]. 

Overview of colonoscopy

The procedure is performed using a colonoscope with a camera attached to its tip and accessory channels that allow the insertion of equipment and fluids to facilitate the procedure. All precautions are taken by all members of the team to ensure a seamless procedure [11].

The person who performs the colonoscopy usually uses his or her right hand to advance or withdraw the scope, while the left hand is used to hold the colonoscope. The operator must be cautious while manipulating the scope, as perforation is a risk while advancing a rigid scope inside the colon [11].

The patient is usually placed in the left lateral decubitus position for the procedure with the patient's legs flexed. The puborectalis and pubococcygeus muscles are relaxed as the legs are bent inward toward the chest. As a result, it is easier to enter through and past the sacral prominence's angle. Specific landmarks can help the clinician know where they are in the colon. Rectum, sacral prominence, tenia coli, splenic flexure, hepatic flexure, and ileocecal valve are important landmarks while advancing or withdrawing the colonoscope. The endoscopist evaluates the mucosa and looks for suspicious lesions as they enter the colon and move toward the cecum. If a polyp is found, it should be removed immediately. The lesion should be tattooed with India ink or methylene blue, and the site should be noted if it is too large to remove. A biopsy should also be taken [11].

A colonoscopy should be done under sedation. It can be performed by the colonoscopist without the assistance of an anesthesiologist or certified registered nurse anesthetist (CRNA); however, this requires the colonoscopist to be knowledgeable about sedative medicines and contraindications. Hence, the colonoscopist needs to be able to divide their attention between the procedure and the patient's comfort and level of sedation [11].

As with any procedure, there are some limitations associated with a colonoscopy, including the extensive bowel preparation, the need for sedation, and the invasiveness of the procedure [5]. Bowel preparation can be time-consuming and uncomfortable, requiring some modifications in medications and food and involving the use of a cleansing agent [5].

Although colonoscopy is a generally safe procedure, the invasive nature of the procedure carries risks. Complications from this procedure include bloating, nausea, and minor gastrointestinal bleeding, which are generally considered minor complications. Others include perforation and hemorrhage, which are the major complications. Of the recommended age group for screening colonoscopy, it has been noted that patients 60 years and above have an increased risk of developing complications [12].

A prospective cohort study carried out by the CDC in five programs revealed that out of 3355 patients who underwent colonoscopies, only eight had major complications (2.38 per 1000 colonoscopies) and warranted hospitalization [12].

Compared with other non-invasive options for CRC screening, the risks and complications associated with this procedure could very well be a reason for the low rate of colonoscopy among African Americans. A further disadvantage is the potential for "interval cancers," which are CRC cases that develop over the extended period between regularly scheduled screening colonoscopies and are estimated to make up 6%-9% of all CRC cases [5].

Compared to other screening modalities (sigmoidoscopy, etc.), colonoscopy can visualize the entire length of the colon and be used therapeutically as it can remove lesions at the time of detection [5]. For this reason, when comparing the financial burden of screening and treatment modalities for CRC, utilizing colonoscopy is the cost-effective option. There has been a reported reduction in the incidence of CRC in patients who undergo colonoscopies. Case-control studies have shown a 31% reduction in CRC-related mortality with colonoscopy and a 53%-72% decline in the incidence of CRC with colonoscopy [5].

Overall, colonoscopy is considered more sensitive than other screening tests. For example, in a study that included 572 asymptomatic individuals who have a family history of CRC, Ng et al. reported that colonoscopy detected 24 advanced adenomas (4.7%) and three CRCs (0.6%) in patients who were reported to be FIT-negative [13].

Trends in colonoscopy

A study demonstrates that the population screened for colon cancer in the United States through colonoscopy between 2000 and 2003 has increased by only 3%. However, the increase was noticed among all the populations except those with non-Medicare low incomes. Hence, Medicare coverage seems to play a vital role in increasing trends in colonoscopy. The same study also demonstrated a decrease in CRC screening in low-income individuals. The increase in colonoscopy utilization was evident in high/middle-income populations [14].

Even though the widespread use of screening colonoscopies may appear to cause an initial rise in incidence due to the identification of early stages of cancer cases, a drop is anticipated over time due to the prevention of CRC by identifying and removing adenomas during screening. 

Recent data show that colonoscopy trends have gone from 20% in 2000 to 61% in 2018 among adults who are 50 years and older. The outcome of such a trend was that many colon and rectal cancer cases could be discovered in earlier stages. For example, the diagnosis of localized stage cancer shifted from 33% in 1995 to 41% in 2005. In contrast, regional stage cancers decreased from 39% to 34% for the same period. Colonoscopy reports have been linked to reducing CRC incidence by 40% and mortality by about 60% [15].

Another study showed that lower gastrointestinal (GI) endoscopy trends in the United States have increased from 48% in 2002 to 53% in 2004 and further to 58% in 2006. Most of the increases were in the age group of 70-74 years. In 2004, the prevalence of lower GI endoscopy among men aged 80 years and older exceeded that among women in the same age group. Conversely, women aged 50-54 years exhibited higher rates of lower GI endoscopy compared to their male counterparts [16].

Studies have shown that while preventive healthcare guidelines recommend regular colon screenings for anyone over 45 years, White people have historically had the highest rate of colonoscopy usage among all racial groups [17].

Latinos and Asians have also completed screenings more than African Americans. Additionally, studies have identified that non-White individuals used FOBT more frequently, while White participants used colonoscopy more frequently for colon cancer screening [18].

The disparity is due to several factors. Lower income levels and restricted access to health insurance have been recognized as socioeconomic barriers to screening among African Americans [19].

Furthermore, discrepancies in access to healthcare facilities, healthcare professionals, and transportation can make it difficult for these individuals to undergo the procedure. Cultural views about cancer, preventive screenings, and distrust in healthcare systems may also contribute to the low utilization rates in African American patients [20].

Factor analysis surrounding utilization of colonoscopy among African Americans

Fear

Studies have identified cancer fear as a barrier and a facilitator for patients to undergo recommended screening colonoscopies [21]. Patients who undergo regular surveillance colonoscopies see it as a choice between "getting cancer" and "having a colonoscopy," with colonoscopy preferred above cancer diagnosis [22].

On the other hand, patients who named cancer fear as a barrier typically reported that they would "prefer not to know" and perceived cancer as a "killer" [21]. Additionally, beliefs, such as the assumption that screening is unnecessary if one is asymptomatic or the fear of obtaining a cancer diagnosis, may influence colonoscopy utilization [21]. Effective communication and education are critical in reducing anxiety and increasing colonoscopy utilization. Fear can be minimized by providing precise and accurate information regarding the procedure, addressing concerns about pain and discomfort, and disputing myths and misconceptions.

A randomized clinical trial conducted by Shahrbabaki et al. showed that patients who received education about colonoscopy procedures experienced lower levels of fear and anxiety than patients who did not. It has been suggested that individuals who had received training prior to their endoscopy felt less anxious [23]. Patients can feel lesser levels of anxiety before a procedure with the help of information, effective communication, complete support from nurses, their emotional awareness, and their ability to address their inaudible inquiries [23]. Nursing counseling significantly reduces the anxiety experienced by patients undergoing an endoscopy. Nurses are more accessible than doctors and have more time for patient education. Nursing care includes education as a key element. They also concluded that patient education fosters therapeutic alliances. Giving patients time to train will boost their confidence and lessen their anxiety and fear [23].

Studies have shown individuals who had a positive colonoscopy experience were less afraid of repeating the procedure and were more likely to choose colonoscopy over other screening methods in the future than individuals who had never experienced the procedure [24]. In cases of first-time patients, the option of sedation was sufficient to allay patients' fears about the discomfort or pain associated with the procedure [21].

**Figure 2:**
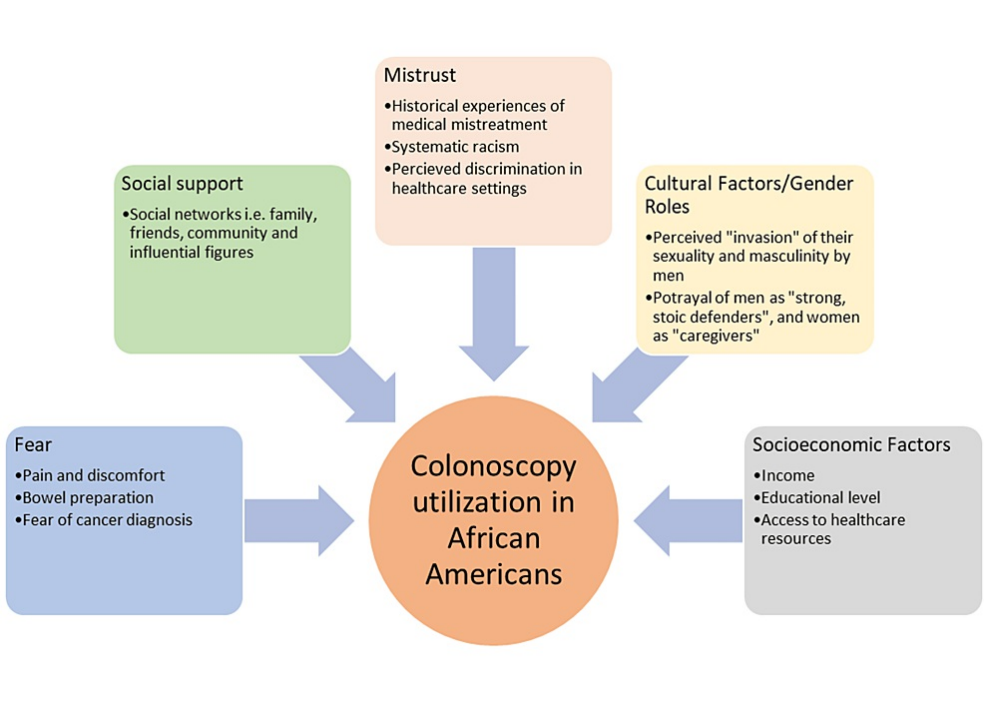
Factors affecting colonoscopy utilization in African Americans. Image credit: Authors.

Culturally sensitive educational initiatives that use a variety of channels, such as community workshops, multimedia platforms, and collaborations with local organizations, can play an essential role in sharing knowledge and lowering fear surrounding colonoscopy. Adequate patient education prior to the procedure can improve confidence and significantly reduce the anxiety and fear associated with colonoscopies (Figure 2).

Social Support

Studies have shown that a lack of supportive connections, social networks, and influential figures can significantly impact African Americans' use of colonoscopies [25]. Research has shown that unmarried individuals have a higher risk of being diagnosed with advanced stage cancer than individuals who are married [26].

Without the assistance and motivation of dependable support systems, colonoscopy-related anxiety and misunderstandings can go untreated and result in reduced screening rates [27]. People may have a distorted perspective of the procedure, emphasizing its possible pain, discomfort during bowel preparation, or complications due to a lack of personal experiences from friends, family, or community leaders [27].

Despite significant evidence regarding its importance, colonoscopy still has a considerable stigma owing to its invasive nature. A solid social support system can help overcome these stigmas, and individuals may feel more comfortable discussing and considering the procedure. For some, the primary care physician is an important influence in the decision to undergo or not undergo a colonoscopy, both in terms of having previous patient education on the importance of colonoscopies and obtaining a referral to have the procedure [28]. Without the support of these sources, many people may not fully understand the significance of colonoscopies in the early identification and prevention of CRC, which would limit their likelihood of going through with the procedure [28].

Understanding the social relationships and support that can influence health behaviors is crucial. While social support is not a cure-all, its importance cannot be overstated. By understanding and leveraging social relationships, we can enhance colonoscopy rates and, as a result, CRC outcomes in African Americans.

Mistrust

The mistrust with colonoscopy utilization can stem from various sources, some of which date as far back as colonial times. Historical experiences of medical mistreatment and systemic racism have generated distrust within the community, exacerbating fear and reluctance to undergo screening [29]. This mistrust is reinforced by anxiety and uncertainty brought on by previous unethical treatment [30].

One of the most infamous medical malpractice cases in American history is the Tuskegee Syphilis Study. The study, which took place between 1932 and 1972, involved withholding necessary medical treatment from African American men with syphilis, which resulted in severe medical consequences and, in many cases, death. Incidents like these have left a legacy of distrust that has influenced attitudes toward many medical procedures, including colonoscopies [31]. 

Due to this history, many African Americans have a longstanding distrust of the medical profession. Mistrust can foster skepticism about the necessity and safety of medical procedures, such as colonoscopies, and result in lower screening rates. The fear of being exploited or receiving substandard care can create a significant barrier to accessing preventative health services.

Aside from historical events, everyday experiences with racial bias and perceived discrimination in healthcare settings contribute to this mistrust. There is a widespread belief that African Americans frequently receive inferior medical care compared to their White counterparts. Notions of such systemic inequities can contribute to the widespread belief that the healthcare system does not serve the best interests of African American patients [31].

The mistrust of African Americans over 50 years toward the medical community is deep-rooted. The implications for colonoscopy usage highlight the broader impact of this distrust in preventative healthcare. Addressing it requires a serious, compassionate, and consistent effort from the medical community and society. Only with a consistent commitment to trust-building can we attempt to bridge the gap in colonoscopy utilization and, as a result, reduce disparities in CRC outcomes among African Americans.

Cultural Factors and Gender Roles

According to research, women are more inclined than men to seek preventative healthcare. In African American communities, where women frequently play significant roles in health decision-making and where doctor-patient relationships are often more established in women than men, women may be more open to having colonoscopies or encouraging their loved ones to undergo the procedure. In contrast, sexual connotation and loss of trust in doctors and the healthcare system were strong barriers for men [32]. A common factor affecting colonoscopy utilization in African American men is the perceived "invasion" of their sexuality and masculinity [33,34].

The procedure includes inserting a scope into the rectum, which can be perceived as invasive and may contradict certain cultural taboos and masculinity perceptions [33]. Some men may perceive the procedure as threatening their dignity or their sense of strength and invulnerability [26]. As a result of these firmly rooted beliefs, patients may be hesitant or refuse to undergo the procedure. Reinforcing this idea, a study by Heslin et al. discovered that men who self-identify as gay or bisexual exhibited a 67% higher likelihood of completing colonoscopies than those identifying as heterosexual [35]. The findings further bring to light the intricate interplay between the norms associated with masculine roles, the decision to undergo invasive screening procedures, and apprehensions about being labeled as gay [36].

Traditional gender roles are highly ingrained in many African American communities. Men are frequently portrayed as strong, stoic defenders, whereas women are portrayed as caregivers [36]. While these roles are evolving, they can affect health perceptions and behaviors, including decisions on preventative healthcare such as colonoscopies. Women are also reported to be aware of the importance of screening in higher numbers than men.

Recognizing the impact of gender roles can assist healthcare providers in tailoring their approach. Providers can establish culturally competent methods that promote colonoscopy utilization by understanding the various challenges and motivators associated with gender. This could include gender-specific patient education materials, personalized messaging, and community engagement campaigns.

Socioeconomic Factors

Socioeconomic factors influence colonoscopy usage among African Americans significantly. Income, education, and access to health insurance can all impact an individual's ability to access and afford preventative healthcare treatments [37].

Individuals with little financial resources may forego or defer elective treatments such as colonoscopies, even if they recognize their importance because medical expenses are a significant concern. Due to conflicting financial demands, many African Americans over 45 years may find a colonoscopy prohibitively expensive. A lower educational level, which is common in some African American communities, may be associated with a lack of understanding regarding the importance of colonoscopies. These individuals may also be less likely to have health insurance or the ability to fund out-of-pocket expenses for a colonoscopy. Individuals in disadvantaged areas may also lack the skill, information, or resources to navigate the healthcare system and book colonoscopies [38].

Furthermore, those in socioeconomically poor areas have less access to healthcare institutions that provide colonoscopies. These places may lack quality healthcare practitioners or clinics suited to perform such screenings, resulting in longer travel times for residents to reach a suitable facility and discouraging them from getting the procedure [37]. 

A study by Lozano et al. determined that unemployment, community insecurity, and high housing burden are the most significant contributors to overall community disadvantage. In that study, over 50% of the overall impact of neighborhood adversity on CRC screening was accounted for by unemployment (38%) and a significant housing burden (16%) [39]. The study by Lozano et al. backs up this claim, arguing that systemic disparities in resource allocation and access to care disproportionately affect African American communities, negatively impacting colonoscopy utilization [39].

Understanding and addressing socioeconomic challenges are crucial in ensuring that all persons, regardless of socioeconomic position, can benefit from critical preventative procedures such as colonoscopies and help improve the rate of colonoscopy utilization in African Americans over 45 years.

Strategies to improve the rate of colonoscopy among African Americans

CRC screening rate remains low among African Americans, despite their increased risk compared to White people [40]. Interventions designed to educate patients about CRC and screening guidelines can improve screening rates and attitudes, and those that contain culturally sensitive materials have also boosted screening among African Americans [41]. Culturally sensitive interventions should focus on dispelling myths, raising awareness about the importance of screening, and addressing anxieties and challenges related to colonoscopy. Multimedia tools, community seminars, and collaborations with local organizations can effectively convey information and educate people to make informed decisions about colonoscopy.

Collaboration among healthcare practitioners, community leaders, and organizations is critical for reaching the African-American population. Community outreach programs should include trusted individuals such as community health workers and faith-based leaders to enhance colonoscopy knowledge and provide support during the screening process. These programs can develop trust, address social determinants of health, and empower individuals to prioritize their colorectal health by forming culturally relevant partnerships [41].

Patient navigation is a proven strategy for increasing CRC screening rates in African Americans and also improves no-show rates and bowel preparation. A randomized trial was conducted in older African Americans to compare the importance of phone navigation and printed material versus printed material alone, and it found a 53% increase in endoscopic screening in the phone navigation group with health-literate subjects showing a stronger effect from navigation [41].

African Americans often belittle their risk of getting CRC, which can be combatted by actively helping them understand their increased risk. As African Americans have been known to hold physician recommendations in high regard, the role of healthcare practitioners in boosting colonoscopy rates among African Americans is critical.

Providing culturally appropriate care, tailored counseling, and strong patient-provider connections is essential. Providers should actively recommend and discuss colonoscopy with eligible patients, emphasizing its benefits and addressing concerns. Reminder systems, like phone calls or text messages, can help highlight the need for screenings and enhance adherence rates [40,41]. 

Addressing the structural barriers to colonoscopy access is critical. Strategies should focus on enhancing transportation options, particularly in underserved areas, to ensure individuals can reach screening facilities. Extending clinic hours and weekend appointments can help individuals with job and family responsibilities. Reduced wait times, language interpretation services, and improved cultural competency training for healthcare professionals can improve the patient experience.

Having identified some of the barriers to CRC screening, including fear, lack of knowledge, lack of provider recommendation, low perceived need for screening, fatalism, mistrust, financial implication, etc., the strategies to improve the rate would tackle the barriers identified. Efforts to increase screening rates among African Americans would take into perspective the barriers noted and tackle each one [40]. The interventions should always be patient-centered and focused on the barriers specific to each patient.

## Conclusions

Despite the proven benefits of colonoscopy in CRC screening, it has remained an underutilized tool among the African American population. Some factors have influenced the rates of colonoscopy utilization, some of which include medical mistrust, fear, patient-doctor relationship, inadequate perception of risk, and negative perception of the screening process. Having explored some of the factors that pose a hindrance to the utilization of colonoscopy among African Americans, some strategies could be implemented and maximized to improve the rate of colonoscopy. It is possible to promote the importance of colonoscopy and increase utilization among African Americans by addressing knowledge gaps, cultural attitudes, financial constraints, and healthcare system flaws. Engaging the community, building trust, and adopting targeted treatments can all help to reduce CRC disparities and improve health outcomes in this population. The major role falls on the physician to improve the physician-patient relationship and the trust between the two parties. Most importantly, each intervention should be patient-centered. More research, collaboration, and implementation of evidence-based initiatives are required for equal access to colonoscopy screenings among all racial and ethnic groups.
